# Transcriptomic and metabolomic profiling of flavonoid biosynthesis provides novel insights into petals coloration in Asian cotton (*Gossypium arboreum* L.)

**DOI:** 10.1186/s12870-022-03800-9

**Published:** 2022-08-30

**Authors:** Aishuang Xing, Xiaoyang Wang, Mian Faisal Nazir, Xiaomeng Zhang, Xiuxiu Wang, Ru Yang, Baojun Chen, Guoyong Fu, Jingjing Wang, Hao Ge, Zhen Peng, Yinhua Jia, Shoupu He, Xiongming Du

**Affiliations:** 1 State Key Laboratory of Cotton Biology, Institute of Cotton Research, Chinese Academy of Agricultural Sciences, Anyang, 455000 China; 2 Zhengzhou Research Base, State Key Laboratory of Cotton Biology, Zhengzhou University, Zhengzhou Henan, 450001 China

**Keywords:** *Gossypium arboreum* L., Petals, Color diversity, RNA-seq, Metabolite, Flavonoid biosynthesis

## Abstract

**Background:**

Asian cotton (*Gossypium arboreum* L.), as a precious germplasm resource of cotton with insect resistance and stress tolerance, possesses a broad spectrum of phenotypic variation related to pigmentation. Flower color affects insect pollination and the ornamental value of plants. Studying flower color of Asian cotton varieties improves the rate of hybridization and thus enriches the diversity of germplasm resources. Meanwhile, it also impacts the development of the horticultural industry. Unfortunately, there is a clear lack of studies concerning intricate mechanisms of cotton flower-color differentiation. Hereby, we report an integrative approach utilizing transcriptome and metabolome concerning flower color variation in three *Gossypium arboreum* cultivars.

**Results:**

A total of 215 differentially accumulated metabolites (DAMs) were identified, including 83 differentially accumulated flavonoids (DAFs). Colorless kaempferol was more abundant in white flowers, while gossypetin-fer showed specificity in white flowers. Quercetin and gossypetin were the main contributors to yellow petal formation. Pelargonidin 3-O-beta-D-glucoside and cyanidin-3-O-(6''-Malonylglucoside) showed high accumulation levels in purple petals. Quercetin and gossypetin pigments also promoted purple flower coloration. Moreover, 8178 differentially expressed genes (DEGs) were identified by RNA sequencing. The correlation results between total anthocyanins and DEGs were explored, indicating that 10 key structural genes and 29 transcription factors promoted anthocyanin biosynthesis and could be candidates for anthocyanin accumulation. Ultimately, we constructed co-expression networks of key DAFs and DEGs and demonstrated the interactions between specific metabolites and transcripts in different color flowers.

**Conclusion:**

This study provides new insights into elucidating the regulatory mechanisms of cotton flower color and lays a potential foundation for generate cotton varieties with highly attractive flowers for pollinators.

**Supplementary Information:**

The online version contains supplementary material available at 10.1186/s12870-022-03800-9.

## Background

Asian cotton (*Gossypium arboreum* L.) is one of the significant sources of natural fiber, initially introduced to China from the Indian subcontinent during the twelfth century [1]. It possesses resilience against biological and environmental stresses and is also a natural source of genetic variation for fiber-related traits [2–5]. Asian cotton has a broad spectrum of phenotypic variation related to pigmentation. The petals are often yellow, yellow–red, purple, or white with and without purplish-red basal spots [6]. There is a long history of research into the color of cotton flowers [7]. The flower color of cotton has also become a tool for genetic and taxonomic studies of cotton [8–10]. Brightly colored petals attract insects like bees and butterflies [11], increasing pollen dispersal, increasing heterosis, and enriching germplasm resources. Accordingly, it is well known that hybridization produces abundant variation. Hybrid cotton has higher fiber yield than inbred cotton [12, 13]. However, the current cotton hybrid breeding is limited by the low pollination rate of natural hybridization [12, 14]. Insect pollination is an effective way of cotton hybrid breeding. Flower color is an important phenotypic trait affecting insect pollination [15, 16]. It is an ideal genetic improvement strategy to generate cotton varieties with highly attractive for pollinator. Moreover, colorful flowers are a significant addition to the horticultural industry [17]. Furthermore, Asian cotton, as a diploid closely related to the *At* genome of upland cotton, has a high-quality gene source. It is relatively easier to realize the characterization of genetic mechanisms than the allotetraploid with complex genomes. Therefore, exploiting cotton flower color has far-reaching implications for the diversity of cotton germplasm resources.

Previously published reports suggested varying levels of co-accumulation of secondary metabolites responsible for plant pigmentations, such as betalains, flavonoids, and carotenoids [18–22]. In general, flavonoids in plants are classified into six groups, chalcones, flavonoids, flavonols, isoflavonoids, anthocyanins, and flavanols [23]. Among them, flavonoids and flavonols are one of the sources of color in fruits and flowers, which are usually yellow or colorless. Quercetin and gossypetin are known flavonoids involved in synthesizing yellow pigments in plants [24–26]. Flavonols like kaempferol, quercetin, and myricetin have important medical properties such as free radical scavenging and antioxidant [27–29]. Anthocyanins are a type of flavonoid that can be found in a variety of plants as a natural water-soluble pigment. They are responsible for the color development of flowers, leaves, and fruits [30–37]. Phenylpropanoid biosynthesis, flavonoid metabolism, and anthocyanin metabolism are three major stages of flavonoid biosynthesis in plants. Flavonoid biosynthesis is aided by the structural genes *PAL*, *C4H*, *4CL*, *CHI*, *CHS*, *F3H*, *DFR*, *ANS*, *UFGT*, and *3GT* [38]. The *F3H* gene was first cloned from Artemisia annua, and it could convert pinealin to dihydrokaempferol by in vitro enzyme activity analysis [39]. In Cheng's study, three *DFR* genes (*DFR1*, *DFR2*, *DFR3*) were cloned from *Ginkgo biloba* and found that *DFR1* could convert dihydroquercetin into colorless anthocyanins, while *DFR2* could convert dihydrokaempferol into white leucopelargonidin [40]. Transcription factors are another type of regulatory gene involved in the phenylpropanoid-flavonoid synthesis pathway. MYB, bHLH, MADS-box, and WD40 are the main identified in the previous study that play a regulatory role in the flavonoid synthesis pathway [41, 42], with MYB, bHLH, and WD40 forming a ternary complex to regulate the expression of structural genes [43]. The bHLH transcription factor and the R2R3-MYB protein activate anthocyanin biosynthetic genes in *Petunia* and most other dicotyledons [44]. Gonzales et al. [45] found that *Arabidopsis* MYB75, MYB90, MYB113 and MYB114 regulate the expression of the *Arabidopsis* anthocyanin biosynthesis genes *F3'H*, *DFR*, *ANS* and *UFGT*.

Advancements in omics have enabled us to integrate multi-omics better to understand regulatory mechanisms behind particular traits [46]. Combining transcriptome and metabonomic methods, Zhang et al. [47] studied the peeling process of winter jujube. They found that a large amount of pigment is deposited in the cell wall, revealing the metabolic pathways and key genes that control the biosynthesis of lignin during the peeling process of winter jujube. Wang et al. [48] emphasized that the red fading of 'Red Bartlett' pears is closely related to the decrease in anthocyanin synthesis, increase in degradation, and inhibition of anthocyanin transport. Another study on Tunisian soft-seed pomegranate [49] identified 51 phenolic compounds, most contained in red–purple pomegranate arils significantly higher than those in light red pomegranate arils. Similarly, the combination of transcriptomic and metabolite approaches, researchers identified VuMYB90-1, VuMYB90-2, VuMYB90-3, VuCPC, VuMYB4, bHLH, and WD40 proteins affecting the accumulation of anthocyanins and flavonoids through regulation structural genes expression [50].

Hybrid cotton varieties produce higher yields than inbred varieties. However, inefficient and costly cross-breeding is a pressing problem in cotton production today. Developing cotton varieties with highly attractive flowers for pollinators is an effective approach to reducing costs and increasing pollination efficiency. This study aimed to decipher petal color variation in Asian cotton utilizing a metabolomics platform coupled with transcriptomics. Our results will provide a genetic basis for flower color variation in Asian cotton, contributing to developing new high-yield hybrid varieties and further enriching cotton germplasm resources.

## Result

### Phenotypes of petals of different Asian cotton varieties

**Fig. 1:**
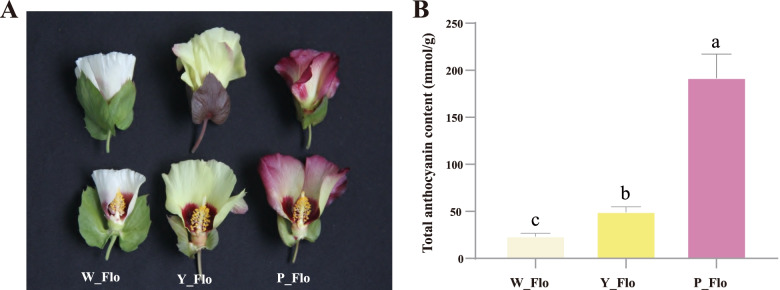
Comparison of phenotype and total anthocyanin content of white, yellow and purple Asiatic cotton (*Gossypium arboreum* L.) petals. **A** Color observation of petals from ‘Shixiya 1’ (W_Flo), ‘GA0146’ (Y_Flo), and ‘GA0149’ (P_Flo) Asiatic cotton. **B** Total anthocyanin content of three *G. arboreum* petals. Letters displayed above each rectangle indicate significant differences between samples (*p* ≤ 0.05)

To comprehend the genetic and metabolite regulatory networks of different flower color varieties, we chose three representative varieties with different petal colors for our study, *Shixiya 1, GA0146, and GA0149*. On the day of anthesis, *Shixiya 1* has a white corolla and dark red petal basal spots and is named W_Flo. The wild-type *GA0146* and its mutant material *GA0149* were biologically similar except for the difference in corolla color (Fig. 1A), with *GA0146* having a yellow corolla and *GA0149* having a purple corolla, named Y_Flo and P_Flo, respectively. We measured the total anthocyanin content of petals for each variety, depicting significant differences in anthocyanin accumulation in petals (Fig. 1B). W_Flo had the lowest anthocyanin content (24.25 mmol/g), while Y_Flo (50.08 mmol/g) and P_Flo (192.53 mmol/g) had significantly higher anthocyanin content.

### Overview of the metabolomic data

**Fig. 2:**
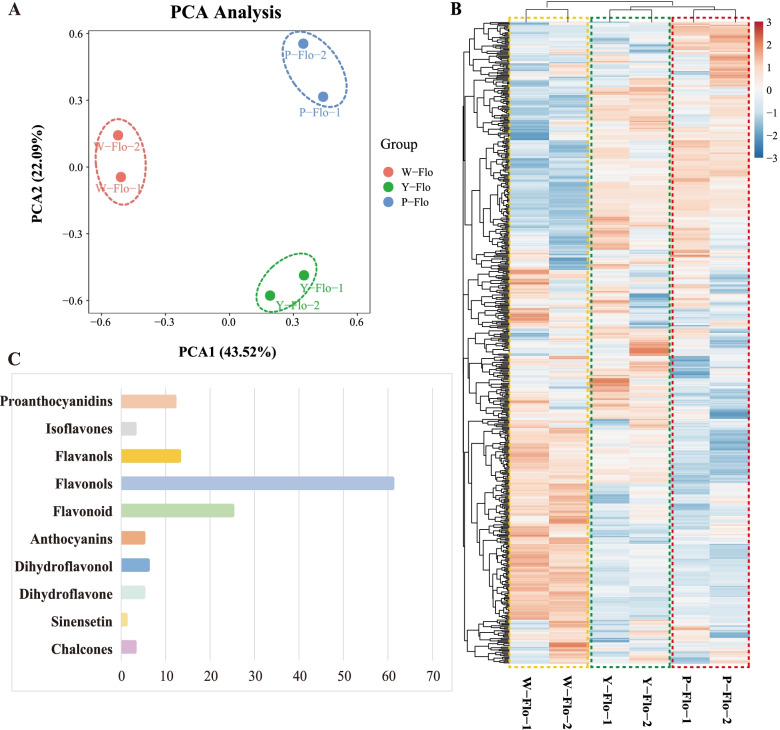
Preliminary analysis of metabolomics data. Comparison of metabolite composition and content in petals. **A** Principal component analysis (PCA) of the metabolite quantification in petals of three colors (W_Flo, Y_Flo, P_Flo). **B** Cluster analysis of all metabolites from samples of W_Flo, Y_Flo and P_Flo. In the heat map, different colors represent the degree of accumulation of each metabolite, with red to blue indicating high to low. **C** Detailed classification of flavonoid metabolites

We determined metabolic profiles using LC–MS/MS to comprehend the differential accumulation of flavonoids and their impact on flower color regulation. Metabolic quantification was subjected to principal component analysis (PCA)(Fig. 2A). The first two components covered 65.61% variation, with 43.52% for PC1 and 22.09% for PC2. We quantified 569 metabolites from 12 major metabolite classes, including flavonoids (122, 21.44%), lipids (78, 13.71%), amino acids and derivatives (76, 13.36%), phenolic acids (60, 10.54%), organic acids (51, 8.96%), nucleotides and derivatives (51, 8.96%), alkaloids (36, 6.33%), tannins (15, 2.64%), lignans and coumarins (9, 1.58%%), steroids (1, 0.18%), terpenoids (1, 0.18%), and others (69, 12.13%) (Fig. S1) ( Additional file 1: Figure S1). The accumulation pattern of identified metabolites in three samples has been presented as a heatmap (Fig. 2B), representing the differential metabolic landscape of different petal colors. Moreover, replicates of each sample were grouped together in both PCA and cluster analysis, emphasizing the quality and reproducibility of the subjected datasets.

Among identified metabolites, we focused on the accumulation pattern of flavonoids. Therefore, we characterized 134 metabolites, including 122 flavonoids and 12 proanthocyanidins (Table S1) (Additional file 2: Table S1). The identified flavonoids could be classified into subclasses, including chalcones, sinensetin, dihydroflavone, anthocyanins, flavonols, flavonoids, isoflavones, and proanthocyanidins (Fig. 2C). Heatmap depicting differential accumulation has been presented as supplementary Fig. S2 (Additional file 3: Figure S2). A significant proportion includes flavonols (61). Moreover, five anthocyanins were identified, including cyanidin-3-O-(6''-Malonylglucoside), pelargonidin 3-O-beta-D-glucoside, cyanidin-3- galactoside chloride, cyanidin 3-glucoside, and cyanidin-O-syringic acid.

**Fig. 3:**
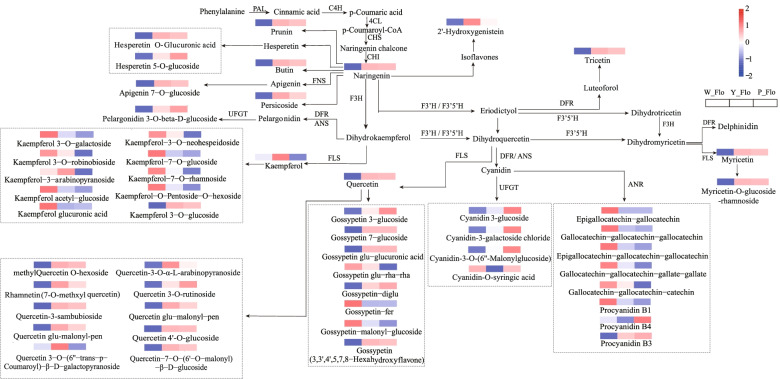
Heat map of phenylpropanoid-flavonoid synthesis pathway of petals of three colors: W_Flo, Y_Flo and P_FLo. The color of each rectangle represents the average amount of flavonoid metabolites in the two biological replicates. The blue rectangle means low and the red means high. PAL: phenyl ammonia-lyase; C4H: cinnamate 4-hydroxylase; 4CL: 4-coumarate-CoA ligase; CHS: chalcone synthase; CHI: chalcone isomerase; DFR: dihydroflavonol 4-reductase; FLS: flavonol synthase; F3H: flavonoid 3-hydroxylase; F3’H: flavonoid 3′-hydroxylase; F3′5’H: flavonoid 3′5′-hydroxylase; FNS, flavonoid synthase; ANS, anthocyanidin synthase; UFGT: UDP-glucose: flavonoid 3- O-glucosyltransferase

We summarized and mapped the phenylpropanoid-flavonoid biosynthetic pathway (Fig. 3). Fifty-four metabolites were identified in the pathway, with their content varying across samples. Interestingly, most kaempferols were relatively high in W_Flo; major quercetin and its derivatives substantially accumulated in Y_Flo; gossypetin species were abundant in Y_Flo and P_Flo; while pelargonidin significantly accumulated in purple petals. In addition to cyanidin-O-syringic acid, three-quarters of cyanidin were highly accumulated in purple petals. The content of catechin derivatives gradually decreased, such as gallocatechin-gallocatechin-catechin (from 175,570(white) to 71,507(purple)). Most of the remaining flavonoid metabolites were high in yellow and purple petals. Therefore, we speculated that kaempferols, quercetin, gossypetin, cyanidin and pelargonidin are key factors in regulating the petal color in Asian cotton. Moreover, pelargonidin and cyanidin may cause the purple corolla. Gossypetin and quercetin are significant yellow pigments involved in the deposition of yellow petal pigments, while kaempferol is an essential metabolite regulating the formation of white petals.

### Metabolic differences among the three colors petals of G. *arboreum*

**Fig. 4:**
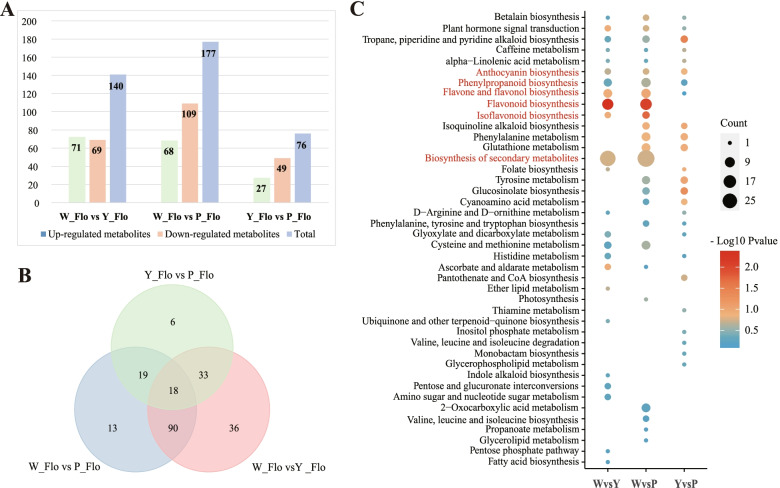
Differentially accumulated metabolites (DAMs) analysis of metabolome. **A** The number of differentially accumulated metabolites between three kinds of petals. **B** Venn diagram showing the overlapping and unique DAMs in comparison groups. **C** The top 25 terms of KEGG enrichment of DAMs. The vertical axis represents the enriched KEGG pathways. The horizontal axis represents the name of comparison groups. The significantly enriched pathways are labeled by red text

Comparisons of metabolic profiles for three samples identified differential accumulation of metabolites with 140, 177, and 76 DAMs in W_Flo vs. Y_Flo, W_Flo vs. P_Flo, and Y_Flo vs. P_Flo, respectively (Fig. 4A and Table S2) (Additional file 4: Table S2). As shown in Fig. 4A, 215 metabolites had differential accumulation in at least one of the compared combinations among the three petals, with up-regulated DAMs varying from 27 to 72 and down-regulated DAMs ranging from 49 to 109. In addition, we identified conserved DAMs between different samples and identified 18 DAMs differentially accumulated in all three samples. These conserved DAMs include amino acids and derivatives, phenolic acids, nucleotides and derivatives, flavonoids, organic acids, and other six categories, with flavonoids being the most abundant class (Fig. 4B). These identified DAMs were mapped to the KEGG pathways for further enrichment analysis. Annotation of DAMs revealed that they are related to anthocyanin biosynthesis, phenylpropanoid biosynthesis, flavone and flavonol biosynthesis, flavonoid biosynthesis, isoflavonoid biosynthesis (Fig. 4C). It means that the metabolites responsible for the different colors of Asian cotton are mainly flavonoids.

We also screened for differential accumulated flavonoids (DAFs) (Table S3) (Additional file 5: Table S3) and found 66, 76 and 21 DAFs were detected in the three comparison groups (W_Flo vs. Y_Flo, W_Flo vs. P_Flo, and Y_Flo vs. P_Flo), respectively. A total of 6 overlapping DAFs were identified in the three comparison groups, including two anthocyanins (pelargonidin 3-O-beta-D-glucoside, cyanidin-3-O-(6''-Malonylglucoside)). Therefore, we speculated that differential accumulation of these anthocyanins may cause a change in cotton flower color. Moreover, in the comparative combination W_Flo vs. Y_Flo and W_Flo vs. P_Flo, as opposed to Y_Flo vs. P_Flo, the phenylpropanoid biosynthesis, flavone and flavonol biosynthesis, flavonoid biosynthesis, isoflavonoid biosynthesis and biosynthesis of secondary metabolites were all more significant. The results suggested that differential accumulation of metabolites in the phenylpropanoid-flavonoid pathway may cause yellow to purple flower color variation. Compared to white and yellow flowers, the DAMs were mainly enriched in isoquinoline alkaloid biosynthesis, phenylalanine metabolism, glutathione metabolism, glucosinolate biosynthesis and cyanoamino acid metabolism pathways, which may result in higher capacity of purple petals than white and yellow flowers in response to external stresses such as pests, oxidation, drought and temperature [51–53].

### Overview of the transcriptome data and identification of DEGs

**Fig. 5:**
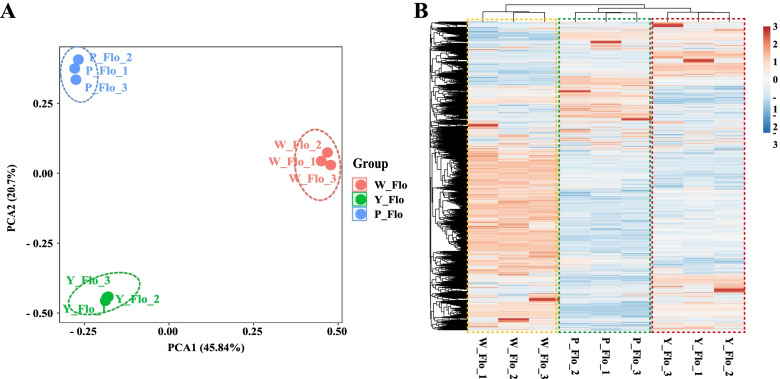
Statistical analysis of transcriptome data from three cotton petal samples. **A** PCA score plot. **B** cluster analysis. The color indicates the relative levels of genes from low (blue) to high (red)

The metabolome is interpreted as the end product of genetic pathways with genes as basic regulators. To identify the genes involved in flower color changes, we further sequenced the transcriptome of Asian cotton petals using RNA-Seq technology to explore the regulatory mechanism of the flavonoid compounds causing flower color changes. We took the petals of W_Flo, Y_Flo and P_Flo on the day of flowering and constructed nine cDNA libraries with three biological replicates in each group. The sequencing yielded a total of 205.55 million reads, and after filtering out the low-quality reads, we obtained a total of 61.39 Gb clean data. The Q30 of each cDNA library was above 92% (Table S4) (Additional file 6: Table S4). We identified 40,960 genes and quantified the expression of these genes in petal tissues. PCA was performed using their FPKM values (Fig. 5A). The replicates in each group clustered together, validating the credibility of transcriptome data sets for further downstream analysis. Moreover, the expression profile based on FPKM values has been presented in Fig. 5B. To verify the accuracy of the transcriptome data, we selected 6 genes from the flavonoid biosynthetic pathway for qRT-PCR expression analysis. The significant positive correlation between RNA-Seq and qRT-PCR data is shown in Fig. S3 (Additional file 7: Figure S3). The relative gene expression level of qRT-PCR was consistent with the FPKM value of the RNA-Seq data, indicating that the RNA-Seq data is credible and accurate.

**Fig. 6:**
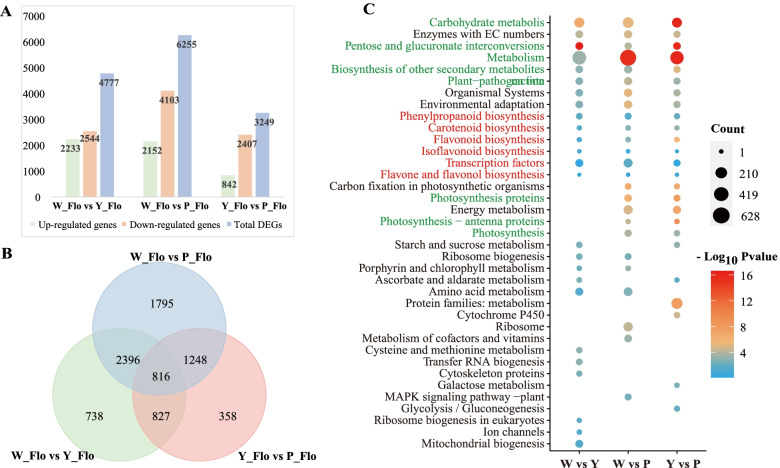
Multivariate statistical analysis of transcriptome data in three Asiatic cotton petals samples. **A** The number of differentially expressed genes in each comparison group. **B** Venn diagram of DEGs in petals of different colors. **C** KEGG enrichment of DEGs in each comparison group. The y-axis indicates the KEGG pathway, and the x-axis indicates the name of each group. Red characters indicate flavonoid synthesis-related pathways and green words show other key metabolic pathways

Using |Log_2_Fold Change|≥ 1, FDR < 0.05, differentially expressed genes of samples with different flower colors were statistically analyzed (Fig. 6). The results showed that there were 4,777 DEGs in the comparison group of W_FLo vs. Y_FLo, in which 2,233 genes were up-regulated, 2,544 genes were down-regulated; in W_FLo vs. P_FLo, there were 6,244 DEGs, including 2,152 genes up-regulated and 4,103 genes down-regulated; there were the fewest number of DEGs (3,249) in ‘Y_FLo vs. P_FLo’ group, with only 842 genes up-regulated and 2,407 genes down-regulated. (Fig. 6A). By comparing the number of DEGs, we hypothesized that more changes in gene expression are required during the change from white petals to yellow or purple than yellow to purple. This is consistent with the peach flesh changes studied by Hong Ying et al. [54]. Furthermore, we identified 816 DEGs as conserved between three groups (Fig. 6B), suggesting their involvement in flower color regulation.

All the 8,178 DEGs (detected in at least one comparative combination) were further annotated using KEGG metabolic pathways and presented in Fig. 6C. It is noteworthy that among the top 25 enriched pathways, we can observe that DEGs are significantly involved in metabolism, carbohydrate metabolism, pentose and glucuronate interconversions, biosynthesis of other secondary metabolites, photosynthesis and plant-pathogen interaction, but a few important genes were also enriched in phenylpropanoid biosynthesis, carotenoid biosynthesis flavonoid biosynthesis, flavone and flavonol (Fig. 6C). Meanwhile, the differential genes are mainly enriched in processes such as photosynthesis and energy metabolism in purple flowers compared to white and yellow flowers. The above results suggest that changes in differential gene expression result in significant changes in metabolic activity. This is similar to the study of the metabolome. To clarify the cellular composition, molecular functions, and biological processes involved in DEGs, we mapped all 8,178 DEGs to the GO database for enrichment analysis (Fig. S4) (Additional file 8: Figure S4). As shown in Figure S4, about 79.13% of the genes had catalytic activity (32.01%, 2,618), transferase activity (12.07%, 987), small molecule binding (11.98%, 980), nucleotide (11.53%, 943) and nucleoside phosphate binding (11.53%, 943). In the cellular component category, about 16.36% of genes constitute the cellular anatomical entity, 8.52% are involved in membrane composition, and more than 22.65% of genes are enriched in biological processes such as macromolecule modification, cellular protein modification process and protein modification process.

### Flavonoid biosynthesis and differential expression of regulatory genes

**Fig. 7:**
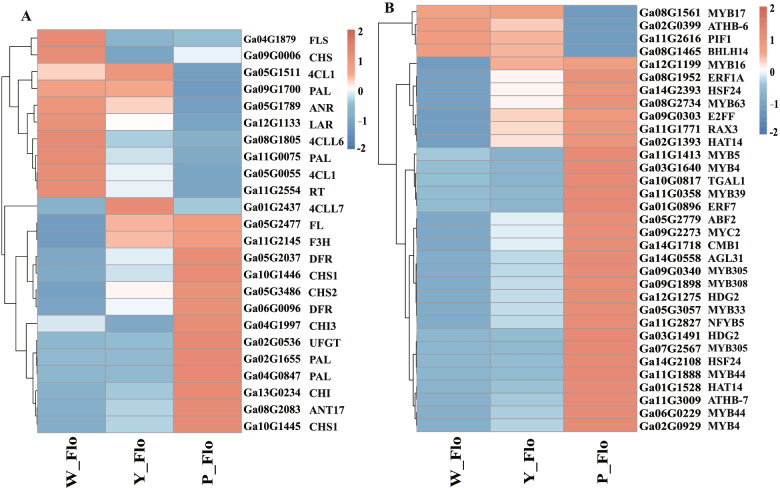
Heatmap showing the expression levels of the differentially expressed structural genes (**A**) and TFs (**B**) associated with color (FPKM value). Orange indicates high expression and blue indicates low expression

Combining the KEGG enrichment results and gene function annotation, we screened important DEGs in the phenylpropanoid-flavonoid synthesis pathway, including the flavonoid biosynthesis (ko00941) pathway, phenylpropanoid biosynthesis (ko00940) pathway, isoflavonoid biosynthesis (ko00943) pathway and flavone and flavonol biosynthesis (ko00944) pathway, with the number of DEGs ranging from 2 to 66. There are 24 key DEGs involved in the flavonoid synthesis pathway potentially associated with color variation, including *4CL* (*Ga05G0055*, *Ga05G1511*, *Ga08G1805*, *Ga01G2437*), *CHI* (*Ga13G0234*, *Ga04G1997*), *CHS* (*Ga09G0006*, *Ga10G1445*, *Ga10G1446*, *Ga05G3486*), *DFR* (*Ga05G2037*, *Ga06G0096*), *F3'H* (*Ga11G2145*), *FL* (*Ga05G2477*, *Ga04G1879*), *LAR* (*Ga12G1133*), *PAL* (*Ga02G1655*, *Ga04G0847*, *Ga09G1700*, *Ga11G0075*), *ANR* (*Ga05G1789*), *ANT17* (*Ga08G2083*), and *UFGT* (*Ga11G2554*, *Ga02G0536*). The expression profile of these genes in different-colored flowers suggested significant variation (Fig. 7A). We calculated Pearson correlation coefficients (Table 1) to identify key genes regulating anthocyanin synthesis to explore the relationship between DEGs and total anthocyanin accumulation. The results showed that 13 DEGs promoted anthocyanin synthesis, and 10 DEGs suppressed anthocyanin synthesis. Among them, the expression levels of *Ga05G2037* (*DFR*), *Ga02G1655* (*PAL*), *Ga05G3486* (*CHS*), *Ga04G0847* (*PAL*), *Ga06G0096* (*DFR*), *Ga10G1446* (*CHS*), *Ga02G0536* (*UFGT*), *Ga13G0234* (*CHI*), *Ga10G1445* (*CHS*) and *Ga08G2083* (*ANT17*) were significantly and positively correlated with the total anthocyanin content (R^2^ ≥ 0.95), suggesting that these 10 genes play an important role in anthocyanin accumulation.

**Table 1:** Differential expression of structural genes in each comparison group

GeneID	Gene Name	Description	Correlation with total anthocyanin	*p*-value	W_Flo	Y_Flo	P_Flo	W_Flo vs Y_Flo			W_Flo vs P_Flo			Y_Flo vs P_Flo		
								**Log**_**2**_**FC**	**Type**	**Fdr**	**Log**_**2**_**FC**	**Type**	**Fdr**	**Log**_**2**_**FC**	**Type**	**Fdr**
Ga09G1700	PAL	Phenylalanine ammonia-lyase	-0.97470	8.27E-06	12.55	12.09	4.93	-	-	-	-	-	-	-1.19	down	2.13E-12
Ga05G1511	4CL1	4-coumarate–CoA ligase 1	-0.83056	5.56E-03	13.28	18.49	5.80	-	-	-	-	-	-	-1.57	down	2.14E-20
Ga05G1789	ANR	Anthocyanidin reductase ((2S)-flavan-3-ol-forming)	-0.81549	7.38E-03	4.73	3.52	1.68	-	-	-	-1.13	down	1.71E-02	-	-	-
Ga12G1133	LAR	Leucoanthocyanidin reductase	-0.80036	9.57E-03	7.61	2.70	0.78	-1.18	down	8.57E-04	-2.93	down	9.05E-11	-1.72	down	7.02E-03
Ga11G2554	RT	Anthocyanidin 3-O-glucosyltransferase	-0.78599	1.20E-02	38.82	14.45	6.44	-1.12	down	1.06E-20	-2.24	down	1.52E-50	-1.08	down	4.49E-08
Ga11G0075	PAL	Phenylalanine ammonia-lyase	-0.75434	1.88E-02	51.24	19.35	11.38	-1.12	down	7.31E-44	-1.82	down	2.61E-81	-	-	-
Ga05G0055	4CL1	4-coumarate–CoA ligase 1	-0.68992	3.97E-02	6.69	0.93	0.21	-2.56	down	8.55E-14	-4.63	down	5.37E-18	-	-	-
Ga08G1805	4CLL6	4-coumarate–CoA ligase-like 6	-0.62496	7.19E-02	3.19	1.44	1.18	-	-	-	-1.07	down	8.31E-03	-	-	-
Ga04G1879	FLS	Flavonol synthase/flavanone 3-hydroxylase	-0.57063	1.09E-01	263.99	92.58	103.27	-1.18	down	3.53E-84	-	-	-	-	-	-
Ga09G0006	CHS	Chalcone synthase	-0.42007	2.60E-01	1.29	0.14	0.37	-2.82	down	1.13E-02	-	-	-	-	-	-
Ga01G2437	4CLL7	4-coumarate–CoA ligase-like 7	-0.25863	5.02E-01	452.81	760.47	492.50	1.04	up	7.39E-250	-	-	-	-	-	-
Ga11G2145	F3H	Naringenin,2-oxoglutarate 3-dioxygenase	0.77845	1.35E-02	28.24	55.01	63.90	1.28	up	2.03E-30	-	-	-	-	-	-
Ga05G2477	FL	Flavonol synthase/flavanone 3-hydroxylase	0.90111	9.08E-04	1.23	30.54	53.94	4.99	up	2.68E-53	-	-	-	-	-	-
Ga04G1997	CHI3	Probable chalcone–flavonone isomerase 3	0.91664	5.07E-04	95.52	64.54	179.41	-	-	-	-	-	-	1.53	up	4.59E-77
Ga05G2037	DFR	Dihydroflavonol 4-reductase	0.97277	1.07E-05	156.36	193.12	264.42	-	-	-	-	-	-	-	-	-
Ga02G1655	PAL	Phenylalanine ammonia-lyase	0.98615	1.02E-06	331.23	330.62	846.62	-	-	-	-	-	-	1.46	up	0.00E + 00
Ga05G3486	CHS2	Chalcone synthase 2	0.98839	5.49E-07	372.03	1298.10	3579.91	2.12	up	0.00E + 00	-	-	-	1.55	up	0.00E + 00
Ga04G0847	PAL	Phenylalanine ammonia-lyase	0.98949	3.87E-07	32.18	33.30	140.68	-	-	-	-	-	-	2.19	up	0.00E + 00
Ga06G0096	DFR	Bifunctional dihydroflavonol 4-reductase/flavanone 4-reductase	0.99094	2.31E-07	164.75	389.70	980.76	1.57	up	1.27E-227	-	-	-	1.41	up	0.00E + 00
Ga10G1446	CHS1	Chalcone synthase 1	0.99131	1.99E-07	328.25	470.70	970.32	-	-	-	-	-	-	1.13	up	1.34E-246
Ga02G0536	UFGT	Anthocyanidin 3-O-glucosyltransferase 2	0.99231	1.31E-07	3950.94	4179.42	11,682.95	-	-	-	-	-	-	1.58	up	0.00E + 00
Ga13G0234	CHI	Chalcone–flavonone isomerase	0.99702	4.77E-09	348.55	421.68	1130.39	-	-	-	-	-	-	1.46	up	6.72E-282
Ga10G1445	CHS1	Chalcone synthase 1	0.99794	1.31E-09	5419.04	7134.36	15,854.65	-	-	-	-	-	-	1.24	up	0.00E + 00
Ga08G2083	ANT17	Leucoanthocyanidin dioxygenase	0.99877	2.17E-10	3130.36	4472.86	14,427.07	-	-	-	-	-	-	1.77	up	0.00E + 00

Transcription factors are a major player in regulating the expression of structural genes and, in turn regulating metabolite synthesis. In this study, we identified and classified 734 TFs, mainly from MYB, AP2/ERF, NF-Y A/B/C, WRKY, and bHLH families (Fig. S5) (Additional file 9: Figure S5). MYB, bZIP, WRKY, and bHLH families play crucial roles in the flavonoid and anthocyanin biosynthetic pathways [45]. These were also differentially expressed in different color petals, where the differences were mainly concentrated in W_Flo vs. P_Flo (144). In brief, both DEGs and TFs were more abundant in white flowers than in purple flowers, explaining why pelargonidin and cyanidin accumulation was highest in P_Flo. In addition, the correlation coefficients between TFs and total anthocyanin content indicated a total of 29 positively regulated TFs (*R*^2^ > 0.9) (Table 2), among which MYB was predominant and might act as a promoter of anthocyanin accumulation. Four negatively regulated (*R*^2^ < 0.9) TFs, including PIF, bHLH, MYB, and ATHB, may be repressors in anthocyanin synthesis. The results showed that the positively regulated transcription factors were expressed at the highest level in P_Flo, while the negative regulators were expressed at the highest level in W_Flo (Fig. 7B).

**Table 2:** Differential expression of known TFS in each comparison group

GeneID	TFs name	Description	Correlation with total anthocyanin	*p*-value	W_Flo	Y_Flo	P_Flo	W_Flo vs Y_Flo			W_Flo vs P_Flo			Y_Flo vs P_Flo		
								**Log**_**2**_**FC**	**Type**	**Fdr**	**Log**_**2**_**FC**	**Type**	**Fdr**	**Log**_**2**_**FC**	**Type**	**Fdr**
Ga11G2616	PIF1	Transcription factor PIF1	-0.96720	2.04E-05	5.35	3.75	0.53	-	-	-	-3.01	down	2.72E-13	-2.75	down	3.95E-10
Ga08G1465	BHLH14	Transcription factor bHLH14	-0.95502	6.08E-05	5.32	3.34	0.23	-	-	-	-4.22	down	9.32E-13	-3.81	down	1.74E-09
Ga08G1561	MYB17	Transcription factor MYB41	-0.93133	2.61E-04	6.69	7.11	0.79	-	-	-	-2.71	down	2.48E-07	-3.10	down	1.15E-09
Ga02G0399	ATHB-6	Homeobox-leucine zipper protein ATHB-6	-0.90599	7.64E-04	17.67	8.53	0.78	-	-	-	-4.16	down	1.05E-23	-3.40	down	4.09E-13
Ga11G0358	MYB39	Transcription factor MYB39	0.90968	6.67E-04	0.19	0.17	1.61	-	-	-	3.50	up	0.000676	3.31	up	0.002633
Ga09G0303	E2FF	E2F transcription factor-like E2FF	0.91693	5.01E-04	4.27	17.38	29.63	2.34	up	6.36E-25	-	-	-	-	-	-
Ga14G2393	HSF24	Heat shock factor protein HSF24	0.92146	4.14E-04	509.37	728.18	949.20	-	-	-	-	-	-	-	-	-
Ga11G1413	MYB5	Transcription repressor MYB5	0.92406	3.69E-04	15.37	13.18	29.76	-	-	-	-	-	-	1.25	up	1.58E-12
Ga05G3057	MYB33	Transcription factor MYB33	0.92628	3.33E-04	8.53	9.82	13.66	-	-	-	1.04	up	2.38E-10	-	-	-
Ga08G2734	MYB63	Transcription factor MYB63	0.93443	2.23E-04	4.00	7.74	12.92	1.29	up	0.000106	2.07	up	6.38E-14	-	-	-
Ga11G1771	RAX3	Transcription factor RAX3	0.94881	9.50E-05	3.79	15.95	32.23	2.40	up	2.12E-23	-	-	-	1.10	up	1.6E-14
Ga05G2779	ABF2	ABSCISIC ACID-INSENSITIVE 5-like protein 5	0.95372	6.71E-05	4.48	6.43	11.08	-	-	-	1.67	up	2.6E-12	-	-	-
Ga09G2273	MYC2	Transcription factor MYC2	0.95869	4.53E-05	10.95	14.05	20.05	-	-	-	-	-	-	-	-	-
Ga03G1640	MYB4	Transcription repressor MYB4	0.96289	3.13E-05	291.38	274.64	452.63	-	-	-	-	-	-	-	-	-
Ga03G1491	HDG2	Homeobox-leucine zipper protein HDG2	0.96443	2.70E-05	29.41	30.06	48.73	-	-	-	-	-	-	-	-	-
Ga01G0896	ERF7	Ethylene-responsive transcription factor 7	0.96582	2.35E-05	105.30	101.73	162.03	-	-	-	-	-	-	-	-	-
Ga14G1718	CMB1	MADS-box protein CMB1	0.96680	2.12E-05	44.14	55.66	76.80	-	-	-	-	-	-	-	-	-
Ga01G1528	HAT14	Homeobox-leucine zipper protein HAT14	0.96717	2.04E-05	0.19	0.28	2.79	-	-	-	4.20	up	5.33E-06	3.41	up	5.02E-05
Ga08G1952	ERF1A	Ethylene-responsive transcription factor 1A	0.96953	1.58E-05	6.05	14.24	28.65	1.59	up	1.26E-08	-	-	-	1.07	up	1.39E-08
Ga14G2108	HSF24	Heat shock factor protein HSF24	0.97291	1.05E-05	190.34	195.66	313.83	-	-	-	-	-	-	-	-	-
Ga10G0817	TGAL1	Transcription factor TGAL1	0.97337	9.88E-06	97.66	90.90	168.75	-	-	-	-	-	-	-	-	-
Ga14G0558	AGL31	Agamous-like MADS-box protein AGL31	0.97482	8.14E-06	0.33	0.57	2.44	-	-	-	3.30	up	0.006616	-	-	-
Ga02G1393	HAT14	Homeobox-leucine zipper protein HAT14	0.98122	2.94E-06	0.05	1.80	12.26	5.19	up	0.000518	7.98	up	2.24E-10	2.84	up	5.25E-14
Ga11G2827	NFYB5	Nuclear transcription factor Y subunit B-5	0.98274	2.19E-06	21.24	32.69	91.42	1.10	up	4.77E-05	-	-	-	1.46	up	1.31E-16
Ga07G2567	MYB305	Myb-related protein 305	0.98479	1.41E-06	461.07	467.90	704.68	-	-	-	-	-	-	-	-	-
Ga12G1199	MYB16	Transcription factor MYB16	0.98595	1.07E-06	0.00	0.69	3.23	-	-	-	7.46	up	1.46E-08	2.30	up	8.97E-05
Ga11G3009	ATHB-7	Homeobox-leucine zipper protein ATHB-7	0.99054	2.69E-07	281.90	314.24	557.30	-	-	-	-	-	-	-	-	-
Ga11G1888	MYB44	Transcription factor MYB44	0.99284	1.02E-07	496.02	515.03	857.18	-	-	-	-	-	-	-	-	-
Ga06G0229	MYB44	Transcription factor MYB44	0.99436	4.42E-08	147.44	171.35	299.22	-	-	-	-	-	-	-	-	-
Ga02G0929	MYB4	Transcription repressor MYB4	0.99587	1.48E-08	35.66	43.98	87.06	-	-	-	-	-	-	1.04	up	5.78E-22
Ga09G1898	MYB308	Myb-related protein 308	0.99615	1.16E-08	32.26	71.78	415.38	1.59	up	2.03E-21	-	-	-	2.53	up	2.1E-196
Ga12G1275	HDG2	Homeobox-leucine zipper protein HDG2	0.99763	2.13E-09	73.08	103.97	224.75	-	-	-	-	-	-	1.22	up	3.4E-235
Ga09G0340	MYB305	Myb-related protein 305	0.99880	1.95E-10	71.57	105.21	281.00	-	-	-	-	-	-	1.45	up	2.64E-77

### Regulatory networks between metabolites and genes associated with flower color

In addition to anthocyanins, quercetin and gossypetin were also considered equally vital substances resulting in yellow petals prior to the characterization of carotenoids [24, 25]. Moreover, quercetin and kaempferol were found to accumulate in high amounts in white *Primula vulgaris* [26]. Therefore, to understand the regulatory relationships between anthocyanins, quercetin, gossypetin, and kaempferol in pigmentation, we performed correlation analysis between key anthocyanins (2), gossypetin (5), quercetin (10), and kaempferol (5) (Table 3), with DEGs in the phenylpropanoid-flavonoid synthesis pathway to construct a network (Table S5) (Additional file 10: Table S5).

**Table 3:** List of the key DAFs regulating flower coloration in the 3 flower types and their Log_2_FC values

Classification	Index	Compounds	W_Flo vs Y_Flo	W_Flo vs P_Flo	Y_Flo vs P_Flo
Gossypetin	Lmmp003271	Gossypetin 7-glucoside	7.021	7.024	-
	Lmmp002529	Gossypetin glu-rha-rha	-	-2.992	-2.194
	mws0855	Gossypetin(3,3',4',5,7,8-Hexahydroxyflavone)	5.947	5.659	-
	Rfg0001-der07	Gossypetin-diglu	2.425	2.932	-
	Rfg0001-der20	Gossypetin-fer	-13.924	-13.924	-
Quercetin	mws1139_N	(2R,3R)-Dihydroquercetin	-2.324	-2.307	-
	pma0214	methylQuercetin O-hexoside	6.546	6.404	-
	Rfg0380-der04	methylquercetin-rut	-1.534	-2.175	-
	pme2954	Quercetin	3.383	3.291	-
	Lmyp004052	Quercetin 3-O-(6''-trans-p-Coumaroyl)-β-D-galactopyranoside	1.265	-	-1.868
	pme3130	Quercetin 4'-O-glucoside	10.117	-	-
	Lmmp003266	Quercetin glu-malonyl-pen	9.649	8.077	-1.572
	mws0045	Quercetin-3-O-α-L-rhamnoside	1.990	2.415	-
	Rfg0376-der08	quercetin-hex-hex	4.193	4.487	-
	pme3369	Rhamnetin (7-O-methxyl quercetin)	2.925	2.212	-
Anthocyanin	pmb0542	Cyanidin-3-O-(6''-Malonylglucoside)	1.273	2.541	1.269
	pme3392	Pelargonidin 3-O-beta-D-glucoside	1.983	3.021	1.038
Kaempferol	Lmmp002130	Kaempferol-rhamnoside-pentaoside-hexaoside	-1.750	-2.480	-
	mws1043_N	kaempferol 3-O-galactoside	-1.471	-2.009	-
	Lmmn003398	Kaempferol acetyl-glucoside	-1.843	-2.305	-
	pme0321	Kaempferol-7-O-rhamnoside	-1.388	-2.573	-1.185
	Hmcp002029	Kaempferol-O-Pentoside-O-hexoside	-1.854	-2.933	-1.079

**Fig. 8:**
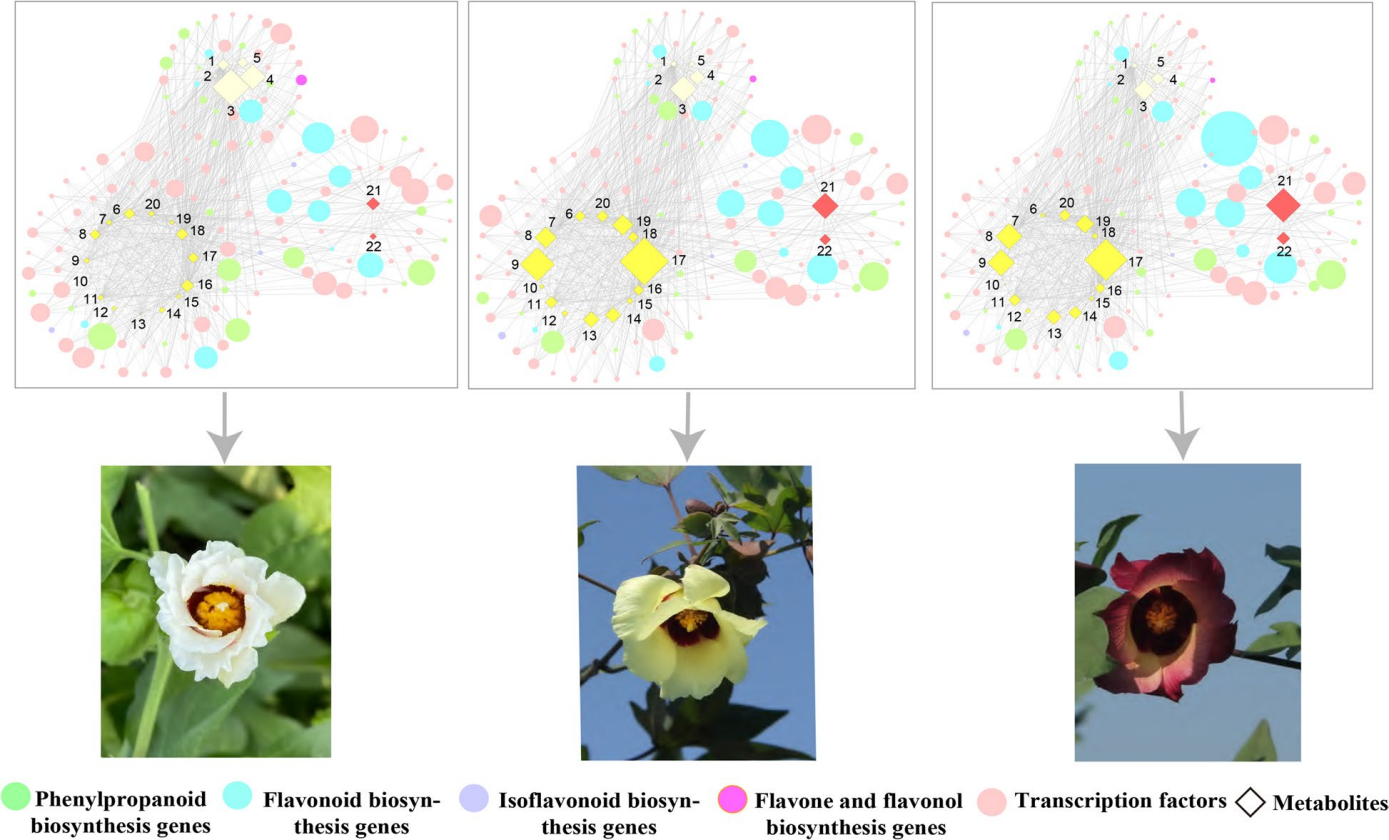
Co-expression networks of differentially expressed genes and metabolites associated with pigmentation. Metabolites including kaempferol, quercetin, gossypiin, and anthocyanins, are shown in milky white, yellow, and pink, respectively. DEGs involve phenylpropanoid biosynthesis, flavonoid biosynthesis, isoflavonoid biosynthesis, flavone and flavonol biosynthesis and transcription factors. The size of the diamond and circle represents the genes FPKM and the metabolite content, respectively. The milk-white diamonds are kaempferol; the yellow diamonds mean quercetin or gossypiin, and the red diamonds are anthocyanins. The names of the metabolites are as follows: 1. Hmcp002029; 2. Lmmp002 l 30; 3. mws 1043-N; 4. Lmmn003398; 5. pme0321; 6. Lmmp002529; 7. Rfg0001-der20; 8. Rfg0376-der08; 9. pma0214; 10. Lmmp003266; 11. Rfg0001-der07; 12. pme3369; 13. pme3130; 14. pme2954; 15. Lmyp004052; 16. Mws1139-N; 17. Lmmpoo3271; 18. Rfg0380-der04; 19. mws0855; 20. mws0045; 21. pme3392; 22. pmb0542. The solid line indicates that genes and metabolites are positively regulated, while the dotted line indicates negative regulation

The regulatory network revealed genes associated with key metabolites and depicted compounds relevant to petal color (Fig. 8). Kaempferol was significantly associated with the white corolla, which is in agreement with the Li’s study. [26]. The colorless kaempferol showed high accumulation levels in W_Flo. Hmcp002029 (kaempferol-O-Pentoside-O-hexoside), pme0321 (kaempferol-7-O-rhamnoside), Lmmn003398 (kaempferol acetyl-glucoside) showed the most significant fold change among the three compared combinations. The expression pattern of two genes (*PAL* (*Ga11G0075*) and *4CL* (*Ga05G0055*)) depicted a significant positive correlation with Hmcp002029 accumulation. Moreover, MYBs, *FL* (*Ga05G2477*) and *F3H* (*Ga11G2145*) jointly negatively regulate the synthesis of pme0321. The genes significantly associated with Lmmn003398 were mainly TFs (MYB and WRKY). The metabolite RFG0001-der20 (gossypetin-fer) was specific in W_Flo, with the value of Log_2_ Fold change up to 13.92. Transcriptome data showed that MYB (*Ga07G1423*, *Ga12G1064*, *Ga11G2161*, *Ga02G1643, Ga03G2432*, *Ga12G1767*), and WERKY (*Ga04G1859*) were highly correlated with Rfg0001-der20. Besides, *Ga05G0055* (*4CL1*) and *Ga04G1879* (*FLS*) were also positively correlated with RFG0001-der20 (*R*^2^ > 0.95). We speculate that these genes positively regulate the synthesis of gossypetin-fer. Previous studies have reported that TFs are essential in the biosynthesis of colorless kaempferol-associated flavonols and cooperate with structural genes to control flower color. Therefore, we hypothesize that MYBs and WRKY are central genes co-expressed or regulated with *4CL*, *FLS*, and *PAL*, thus responsible for forming the white corolla. For Y_Flo (Fig. 8), the results showed a significantly higher accumulation of quercetin and gossypetin compared to white flowers, especially pme3130 (Log_2_ FC = 10.12), Lmmp003266 (Log_2_FC = 9.65), Lmmp003271 (Log_2_ FC = 7.02), and pma0214 (Log_2_ FC = 6.55). Interestingly, *Ga05G0055* (*4CL1*) and *Ga04G1879* (*FLS*) were negatively correlated with Lmmp003271, pme3130, and pma0214, with high expression of these two genes, suppressed gossypetin 7-glucoside, quercetin 4'-O-glucoside, and methylquercetin O-hexoside synthesis. Although Lmmp003266 accumulated only in trace amounts in Y_Flo, it was significantly higher in yellow flowers than in white and purple flowers. Some members of the bHLH, MYB, and WERKY family, such as MYB12, MYB30, MYB306, MYB1236, MYB44, MYB61, bHLH14, WRKY2, were expressed and had the lowest expression in Y_Flo but the highest content of quercetin or gossypetin, which are responsible for yellow petals, are negatively regulated by these TFs. The co-expression network **(**Fig. 8**)** showed that multiple metabolites regulated the purple petal species, and four kinds of metabolites were present in the purple flower species. The contents of pma3392 and pmb0542 gradually increased in the three groups, with the purple petals having the highest abundance, indicating they are essential for purple petals. Pma3392 accumulation not only relied on the positive regulation of *CHS* (*Ga10G1445*, *Ga10G1446*, *Ga05G3486*), *DFR* (*Ga06G0096*, *Ga05G2037*), *FL* (*Ga05G2477*), and MYB (*Ga09G0340*, *Ga05G2658*, *Ga02G0929*, *Ga09G0647*, *Ga08G2734*) but was also negatively regulated by bHLH (*Ga08G1465*). Pmb0542 was mainly regulated by MYB, where MYB4 (*Ga03G2139*) positively regulated the accumulation of pmb0542, while MYB16 (*Ga04G1608*) negatively regulated the synthesis of pmb0542.

In general, the purple petals appeared to be formed by the differential accumulation of anthocyanins, quercetin and are influenced by several metabolites. The yellow petals seemed to be most influenced by gossypetin and quercetin. On the other hand, the white petals resulted from the weak accumulation of Rfg0001-der20 and the high kaempferol content.

## Discussions

The flower color in Asian cotton ranges from white to yellow to purple. Brightly colored petals attract insects, spreading pollen and enriching Asian cotton's germplasm resources. Asian cotton can also be used as ornamental due to its eye-appealing flower characteristics. Moreover, flavonoids are involved in forming and developing flowers, fruits, and seeds in plants and other functions such as antioxidant activity, UV protection, and protection against biotic and abiotic stresses. The study of flavonoids in cotton petals could provide a new way for healthy anthocyanin extraction and thus increasing the value-addition of Asian cotton. To date, metabolome concerning flower petals in Asian cotton has not been reported. In this study, we systematically implied metabolomics and transcriptomics approaches to identify the major flavonoid metabolites affecting Asian cotton petals and screened key genes associated with petal color formation, providing important information for the enrichment of cotton germplasm resources.

### The important role of anthocyanins in the color formation of Asian cotton petals

Anthocyanins are the primary factor influencing the coloration of different plant organs [34–37, 55, 56]. Several studies have demonstrated the essential role of anthocyanins in color formation. For instance, Xue et al. [34] depicted association of anthocyanins with red-colored seed-coat in peanut. A study by Qiu et al. concerning passion fruit [57] found that the total anthocyanin content of purple fruit was significantly higher than that of yellow fruit, raising the potential value of passion fruit as a functional food. Purple wheat [58] has special health benefits, with the color of purple wheat seeds deepening and the total anthocyanin content increasing over time during the developmental stages of purple wheat. White, yellow, blue and pink *Primula vulgaris* [26] showed a gradual increase in total anthocyanin content as the color deepened. Similarly, Asian cotton petals are rich in anthocyanins, so they can also be used as a natural source of pigments in food. Through phenotypically identifying and comparing the total anthocyanin content of different varieties of Asian cotton, we found that the total anthocyanin content of white, yellow, and purple petals was significantly different. Therefore, purple petals can be used as a natural source of anthocyanins to enhance the added value of Asian cotton.

### The potential metabolites involved in flower coloration

The determination of secondary metabolite content and species has an essential function in the research exploitation of species. Flavonoids are vital secondary metabolites in plants [59, 60]. During plant growth and development, flavonoids fulfill a variety of physiological functions. They changed the color of plant organs [61], defended against biological attacks such as pathogenic bacteria [62], and inhibited the harm of abiotic factors such as temperature, drought, and salinity [55, 63–65]. Therefore, we performed a qualitative and quantitative analysis of secondary metabolites in Asian cotton petals. A total of 134 flavonoids were identified by metabolomic analysis. Forty-eight metabolite components (including 32 DAFs) of flavonoids, flavanols and anthocyanins were enriched in the well-known metabolic pathways. According to the analysis of W_Flo, Y_Flo, and P_Flo flavonoid compounds, we found a dramatic shift in the content of metabolites in the phenylpropanoid flavonoid biosynthetic pathway along with the deepening of petal color, with a gradual decrease in the content of catechin derivatives such as gallocatechin. Therefore, we speculated that catechins are necessary precursors for synthesizing anthocyanins, consistent with the results of Wang et al. [58] on purple wheat seeds. Moreover, other flavonoid metabolites showed multiple trends, indicating that Asian cotton petal color regulation is a complex and dynamic process regulated by multiple factors.

More than 20 kinds of anthocyanin have been identified so far, among which cornflower, geranium, paeoniflorin, petunia pigments, ariocyanin and mallow pigments are more common in plants [66]. In this study, two key anthocyanins were identified: cyanidin and pelargonidin. Cyanidin causes red–purple color variation, while pelargonidin contributes to orange and red [67]. Pelargonidin 3-O-beta-D-glucoside and cyanidin -3-O-(6''-Malonylglucoside) depicted differential accumulation in all three petals. Xue et al. [68] emphasized the regulatory role of cyanidin and pelargonidin in strawberry flower color, resulting in darkening of flower color. Quercetin and gossypetin were considered a major yellow pigment when carotenoids were not found in *P. vulgaris* [24, 25]. By analyzing the phenylpropanoid-flavonoid biosynthetic pathway, we identified 12 quercetin and 8 gossypetin that differed significantly in different color petals. Quercetin 4'-O-glucoside, methylquercetin O-hexoside, gossypetin 7-glucoside, and quercetin glu-malonyl-pen were specific in the yellow group, and we predicted them to be the main metabolites responsible for yellow petals. Although anthocyanin components were detected in W_Flo, they may be due to the effect of floral base spots. However, the white petals contained higher levels of kaempferol than the other two groups, especially kaempferol acetyl-glucoside and kaempferol 3-O-galactoside had the highest content, and kaempferol-O-pentoside-O-hexoside had the highest foldchange, which may be an important factor in regulating the pigmentation of white petals.

### The key DEGs responsible for the phenylpropanoid – flavonoid biosynthesis pathway

Transcriptome analysis is important for identifying genes responsible for a stage-specific trait. The analysis of different comparison groups revealed that only a few genes showed an up-regulation trend in expression as petal color deepened, suggesting that the process of color deepening requires only a few key involvements. Moreover, the functional enrichment of DEGs indicated that changes in differential gene expression caused significant changes in metabolic activities. Some key secondary metabolites in plants have defensive functions and chemosensory effects against pests and diseases [69, 70]. However, we also found that anthocyanin biosynthesis is linked to the phenylpropanoid-flavonoid synthesis pathway, carbohydrate metabolism, pentose and glucuronate interconversions, and plant-pathogen interaction. Sugar, carbohydrate, and plant hormone play a crucial role in anthocyanin biosynthesis [58]. We also screened important DEGs in the phenylpropanoid flavonoid synthesis pathway by analyzing the transcriptome, including *4CL*, *CHI*, *CHS*, *DFR*, *F3'H*, *FL*, *LAR*, *PAL*, and *UFGT*, and most of these structural genes were significantly expressed in purple petals [71]. We further selected 10 structural genes that were notably associated with the total anthocyanin content, suggesting critical roles of *DFR*, *PAL*, *CHS*, *UFGT* and *CHI* in accumulating anthocyanins [72]. We also identified MYB that may act as promoters of anthocyanin accumulation; *Ga11G2616*(PIF), *Ga08G1465*(bHLH), *Ga08G1561*(MYB) and *Ga02G0399*(ATHB) that may act as repressors of anthocyanin synthesis [73–75]. These results suggest that these 10 structural genes and 29 TFs are candidate genes responsible for regulating petal color traits.

### Co-expression networks of key metabolites and genes

The combined transcriptome and metabolome analysis is an important tool for identifying the genes responsible for the different petal colors [48, 76, 77]. Correlation analysis between metabolome and transcriptome showed that the expression levels of some DEGs were closely related to the accumulation of flavonoid metabolites. Therefore, we constructed an interaction network containing 161 DEGs involved in the phenylpropanoid-flavonoid synthesis pathway and 29 flavonoids (7 kaempferol, 10 quercetin, 5 gossypetin, and 2 anthocyanins). Overall, purple petals were regulated by multiple metabolites, with kaempferol, quercetin gossypetin and anthocyanins all present in the purple petals. Pelargonidin 3-O-beta-D-glucoside and cyanidin-3-O-(6''-malonylglucoside) are highly accumulated in P_Flo and are essential for the formation of purple petals. Combined with the correlation analysis, *CHS* and *DFR* were considered as key structural genes in the flavonoid biosynthesis pathway in purple petals [78–80]. As we all know, *CHS* is a firmware rate-limiting enzyme of flavonoid biosynthesis that affects downstream secondary metabolites, while *DFR* is a key enzyme in the anthocyanin synthesis pathway that controls the accumulation of colorless anthocyanin-like metabolites in plants [19, 81]. These results suggest that the expression of flavonoid biosynthetic genes contributes to the accumulation of anthocyanins in purple petal forming. Our findings show that white petals are formed because of the weak accumulation of gossypetin-fer and high levels of kaempferol-7-O-glucoside, kaempferol 3-O-galactoside, and kaempferol acetyl-glucoside. These kaempferol compounds are synthesized in MYBs and bHLH co-expressed with *4CL* and *PAL* resulted. It is consistent with the study of *P. vulgaris* [26]. Yellow petals are regulated by most gossypetin, mainly negatively regulated by *4CL*, *FLS*, MYB, bHLH, and WERKY. Surprisingly, the co-expression network of Lmmp003271 (gossypetin 7-glucoside) indicated that *Ga05G0055* (*4CL1*) had negative regulation of its accumulation, but this gene also positively regulated the synthesis of Rfg0001-der20 (gossypetin-fer). It implies that the low expression of *Ga05G0055* increases the accumulation of gossypetin 7-glucoside and inhibits the synthesis of gossypetin-fer.

## Conclusions

In the present study, we combined transcriptomic and metabolomic analyses to elucidate the mechanism of color differences between different Asian cotton varieties on a flowering day. A total of 122 flavonoid metabolites (including 5 anthocyanins) were identified in the three groups of samples. Moreover, we summarized the important metabolites and genes in the phenylpropanoid-flavonoid synthesis pathway, key factors intimately associated with petal color. We preliminarily screened for DEGs significantly associated with the phenylpropanoid-flavonoid synthesis pathway and, more importantly, identified transcription factors positively and negatively regulating anthocyanin content. In addition, 10 DEGs and 29 TFs played vital roles in anthocyanin accumulation. Ultimately, color-associated co-expression networks were constructed by joint analysis to screen metabolites and candidate genes associated with different petal colors. We have elucidated the metabolites and major genes that regulate Asian cotton petal color through comparative and integrative analyses, providing a basic framework and key basis for further studies on cotton flower color. However, further studies on these candidate metabolites and genes are needed to clarify the molecular mechanisms.

## Materials and methods

### Plant material

Three varieties of Asian cotton with different colored petals, *Shixiya1* (white), *GA0146* (yellow) and *GA0149* (purple), were planted in three rows at the experimental base of the Institute of Cotton, Chinese Academy of Agricultural Sciences, Anyang, Henan Province (36°07′ N, 114°50′ E). Five completely open flowers from each row on the same day were taken for phenotype observation and their petals for total anthocyanin and RNA extraction. All samples were frozen in liquid nitrogen immediately after sampling and stored immediately in a -80 °C refrigerator.

### Measurement of total anthocyanin content

The method of extracting total anthocyanins from *Camellia sinensis* petals by Fu et al. [82] was used and the extraction conditions were optimized. Asian cotton petals were ground into powder in liquid nitrogen. About 0.65 g of dry powder was added to 20 ml of 95% (0.1 mol L^−1^ HCL) ethanol solution and then heated in a water bath at 60 °C for two hours. Finally, the absorbance values of the extracts were measured by the BioTek microplate reader (Gene Company Limited, America) at 520 nm, 620 nm and 650 nm. The total anthocyanin content was calculated by the formula: A = (A530-A620)-0.1(A650-A620), anthocyanin content (mmol/g FW) = A × V × 1,000/489.72 m, where V represents the volume of the extract and m represents the weight of the dried petal powder. Ninety-five percent (0.1 mol L^−1^ HCL) ethanol solution was used as the blank control. Three replicates were analyzed for each sample.

### Metabolite identification and quantification

A series of metabolite extraction, identification and quantification procedures were carried out at Wuhan Metware Biotechnology Co., Ltd (https://www.metware.cn) following the company's standard procedures [83, 84]. Cryo-preserved samples were weighed and extracted with 1.0 ml of 70% methanol at 4 °C. Extracts were analyzed using liquid chromatography-mass spectrometry/M.S. analysis (LC–MS/MS, UPLC, Shim-pack UFLC SHIMADZU CBM30A system; MS, Applied Biosystems 6500 QTRAP). All metabolites were identified and quantified by Metware's metabolite database and public metabolite database. Differential accumulation of metabolites (DAMs) between samples was identified using orthogonal partial least squares discriminant analysis. Metabolites with |Log_2_ Foldchange|≥ 1 and VIP (variable importance in project) ≥ 1 were defined as DAMs.

### Transcriptome sequencing and analysis of Asian cotton flower

Total RNA was extracted from the samples (*Shixiya 1*, *GA0146* and *GA0149*) with the RNA Extraction kit (TIANGEN, Beijing, China). The RNA quality and concentration were assessed with agarose gel electrophoresis and NanoDrop2000 spectrophotometer. RNA sample quality testing, library construction and sequencing for each sample were done at Biomarker Biotechnology (http://www.biomarker.com.cn). The cDNA libraries were sequenced on an Illumina NovaSeq 6,000 platform with generating paired-end reads. Low-quality data containing adapter and poly-N were removed for downstream analysis. The resulting set of high-quality clean reads was used for transcriptome analysis. The clean reads were localized to the Asian cotton reference genome (CRI-v1.0) (https://www.cottongen.org/data/download) [85] using Hisat2 to obtain unigenes. Fragments per kilobase of exon model per million mapped fragments (FPKM) for all genes to determine gene expression values. The differentially expressed genes (DEGs) were identified by R package DESeq2 for subsequent analysis. The genes featuring FDR < 0.05 and |Log_2_ Fold change|≥ 1 were considered DEGs. The Gene Ontology (GO) and Kyoto Encyclopedia of Genes and Genomes (KEGG) databases were applied further to annotate the identified DEGs from each pairwise combination using TBtools software [86]. Taking q-value < 0.05 as the criterion, twenty-five significant pathways were selected. Heat maps were generated using the OmicStudio tools at https://www.omicstudio.cn/tool.

Considering the important role of phenylpropanoid-flavonoid pathway structural genes and TFs in anthocyanin synthesis, the related structural genes and TFs in all samples were identified. Combined with KEGG enrichment results and transcription annotation files, all the annotated TFs were retrieved by searching the transcription annotation file. The related structural genes were filtered in ko00940, ko00941, ko00943 and ko00944 pathways. The Pearson correlation coefficients (*R*^2^) between key genes in the phenylpropanoid-flavonoid pathway and the total anthocyanin content of each sample were calculated using the R package Hmisc [87]. The TFs with |*R*^2^|> 0.9 and the structural genes with |*R*^2^|> 0.95 were retained for subsequent analysis.

### Integrative analysis of key metabolites and genes

To analyze the interactions between genes and metabolites related to flower coloration, we constructed a co-expression network between key DAMs and important DEGs. First, we calculated the mean of key DAMs content and important DEGs expression of three biological replicates in each sample. Then, R package Hmisc was used to calculate the correlation coefficient between metabolites and genes. The DAMs and DEGs between three colored cotton were selected when |*R*^2^|> 0.8. Finally, co-expression networks were constructed using the correlation coefficients of genes and metabolites to reveal the interactions between petal color metabolites and genes. The co-expression networks were visualized using Cytoscape software (version 3.8.2).

### The quantitative real time-PCR validation

Total RNA was reverse-transcribed in a 20 μL reaction mixture using the HiScript® II Q RT SuperMix for qPCR (+ gDNA wiper) kit (Vazyme, China). The PCR product was examined by 1.2% agarose gel (Tsingke, China) electrophoresis. If the band size and brightness met the requirements, the cDNA was of good quality and could be used for qRT-PCR experiments. The 20 μL reactions were performed with 10 μL of ChamQTM SYBR® qPCR Master Mix (High ROX Premixed), 1.0 μL 10 mM forward and reverse primers, 7.0 μL of ddH2O and 2.0 μL 5 times diluted cDNA template. The cotton Histone3 was used as an internal reference gene. The ABI Prism 7500 Fast system was used to perform qRT-PCR. The primers used for qRT-PCR are listed in Table S6 (Additional file 11: Table S6). The relative expression of genes was calculated by the 2^−ΔΔCT^ method [88].

## Supplementary Information

**Additionalfile 1: Figure S1. **Classification and statistics of all metabolites obtained.

**Additionalfile 2: Table S1. **The classification and quantification results of all flavonoid metabolites.

**Additionalfile 3: Figure S2.** The heatmap analysis of allflavonoid metabolites by TBtools. A: chalcones;B: sinensetin; C: dihydroflavone; D: dihydroflavonol; E: anthocyanins; F:flavonoid; G: flavonols; H: flavanols; I: isoflavones; J: proanthocyanidins; I:flavonoid; II: Tannins.

**Additionalfile 4: Table S2. **All differentially expressed genes in each comparison group.

**Additionalfile 5: Table S3. **Differentially accumulated flavonoid metabolites in each comparison group.

**Additional file 6: Table S4.** Overview of the transcriptome sequencing dataset.

**Additionalfile 7: Figure S3. **qRT-PCR validation of gene expression level in the transcriptome.

**Additionalfile 8: Figure S4. **The enrichment results of all differentially expressed genes.

**Additionalfile 9: Figure S5. **The identification and classification results of all TFs.

**Additionalfile 10: Table S5. **The critical color-related metabolites and genes in co-expression networks.

**Additionalfile 11: Table S6. **The primers for the qRT-PCR used in this study.

## Data Availability

The datasets generated and analyzed in the current study are available from the corresponding author on reasonable request. All data generated or analyzed during this study are included in this published article and its supplementary information files. The raw RNA-seq data are freely available at NCBI project PRJNA854799.

## Abbreviations

*4CL*: 4-Coumarate-CoA ligase
*ANS*: Anthocyanidin synthase
*C4H*: Cinnamate 4-hydroxylase
*CHI*: Chalcone isomerase
*CHS*: Chalcone synthase
*DAF*: Differentially accumulated flavonoid metabolite
DAM: Differentially accumulated metabolite
DEG: Differential expressed gene
*DFR*: Dihydroflavonol 4-reductase
*F3**′**5’H*: Flavonoid 3′5′-hydroxylase
*F3H*: Flavonoid 3-hydroxylase
*F3'H*: Flavonoid 3′-hydroxylase
*FLS*: Flavonol synthase
*FNS*: Flavonoid synthase
FPKM: Fragments Per Kilobase of exon model per Million mapped fragments
GO: Gene Ontology
KEGG: Kyoto Encyclopedia of Genes and Genomes
*LAR*: Leucoanthocyantin reductase
*PAL*: Phenyl ammonia-lyase
PCA: Principal component analysis
TF: Transcription factor
*UFGT(3GT)*: UDP-glucose: flavonoid 3-O-glucosyltransferase
