# Supplementary figures and images for: Transcriptomic and metabolomic profiling of flavonoid biosynthesis provides novel insights into petals coloration in Asian cotton (Gossypium arboreum L.)
Source: BMC Plant Biol. 2022 Aug 30;22:416. doi: 10.1186/s12870-022-03800-9 (PMC9425979; doi:10.1186/s12870-022-03800-9)

**Figure S1**

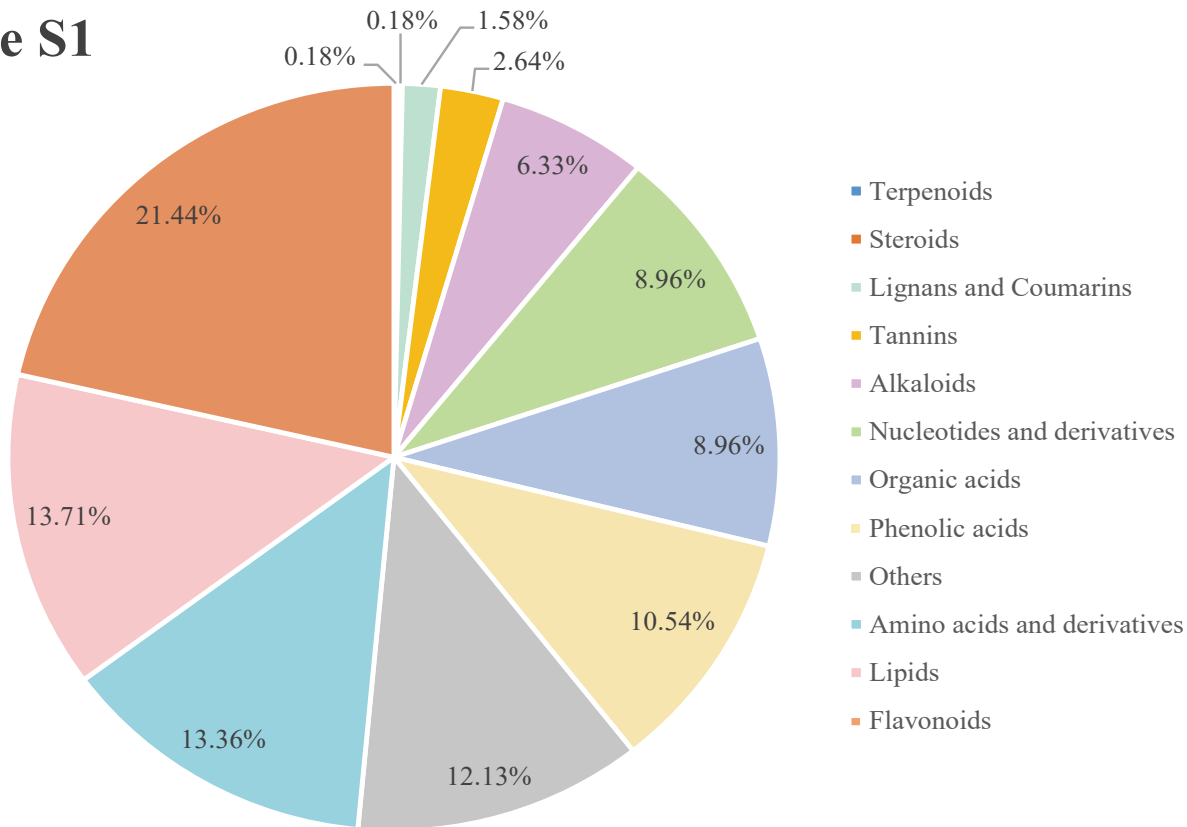

Supplement: Supplementary file 1 — Additionalfile 1: Figure S1. Classification and statistics of all metabolites obtained. [file 12870_2022_3800_MOESM1_ESM.pdf]

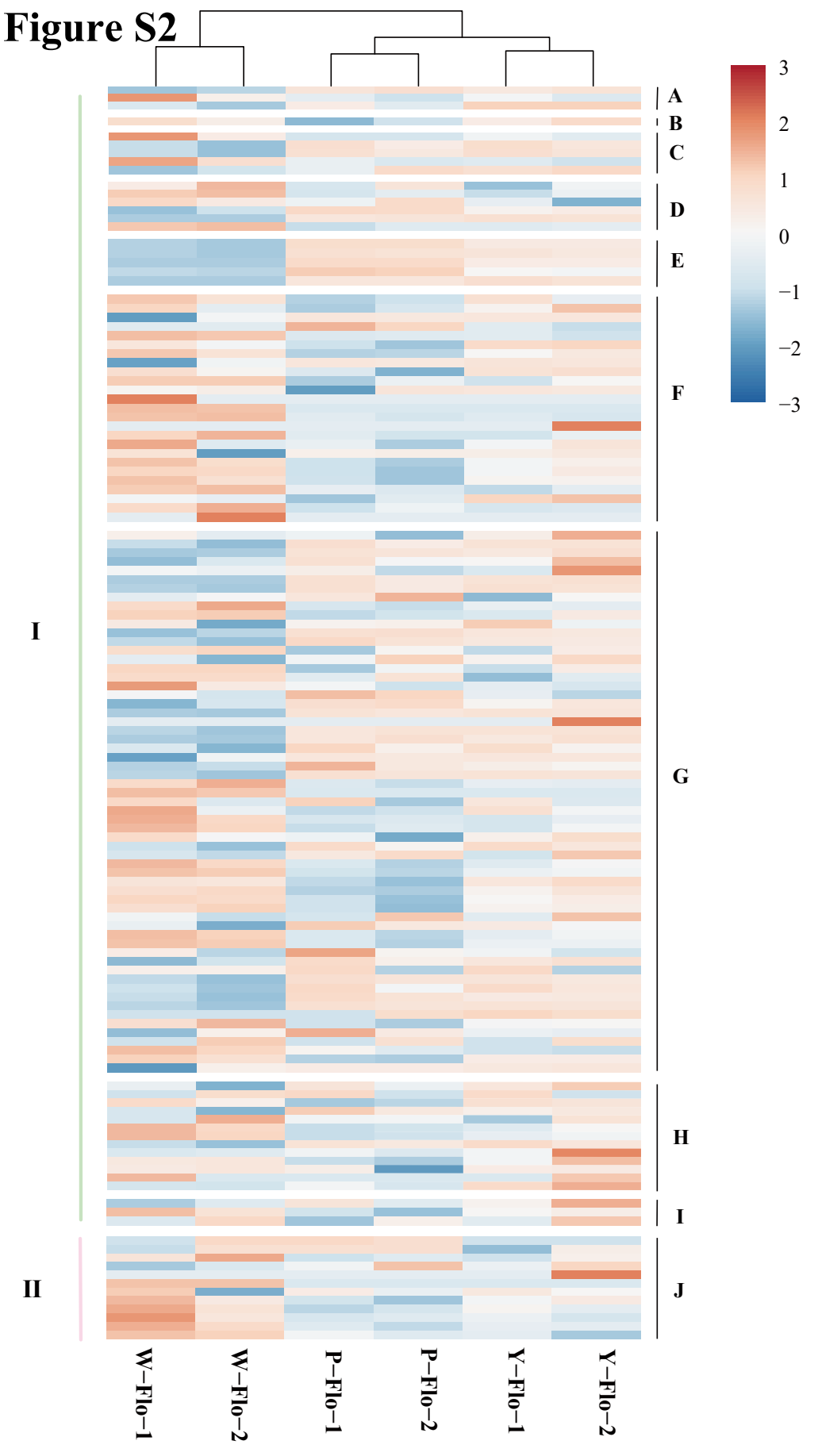

Supplement: Supplementary file 3 — Additionalfile 3: Figure S2. The heatmap analysis of allflavonoid metabolites by TBtools. A: chalcones;B: sinensetin; C: dihydroflavone; D: dihydroflavonol; E: anthocyanins; F:flavonoid; G: flavonols; H: flavanols; I: isoflavones; J: proanthocyanidins; I:flavonoid; II: Tannins. [file 12870_2022_3800_MOESM3_ESM.pdf]

# Figure S3

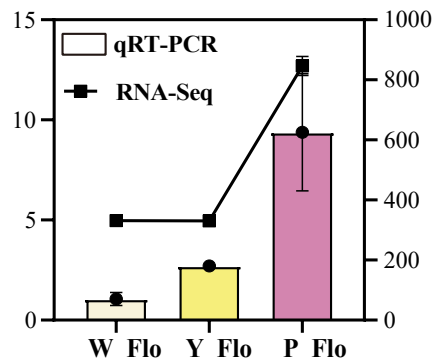

Ga02G1655

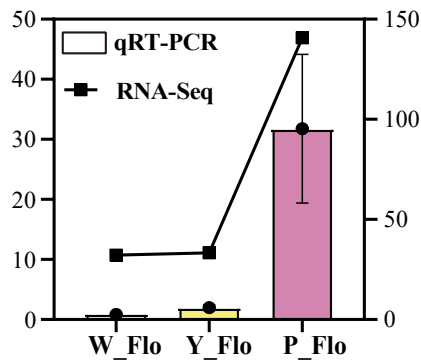

Ga04G0847

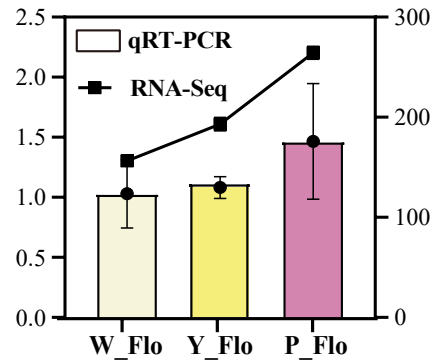

Ga05G2037

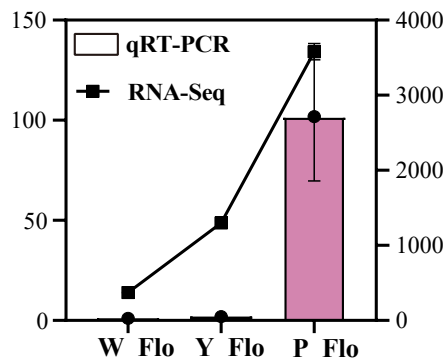

Ga05G3486

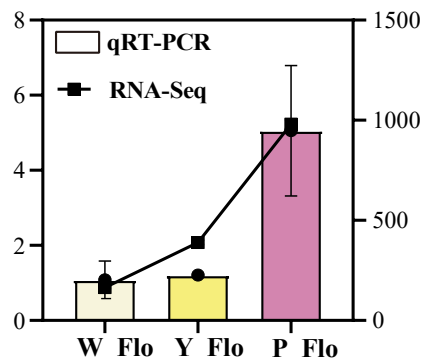

Ga06G0096

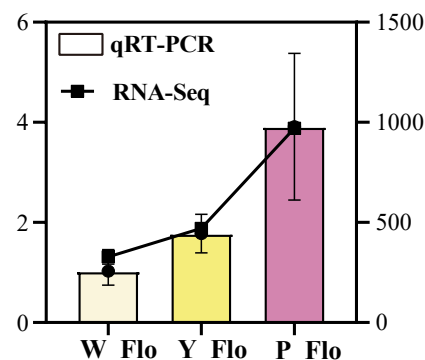

Ga10G1446

Supplement: Supplementary file 7 — Additionalfile 7: Figure S3. qRT-PCR validation of gene expression level in the transcriptome. [file 12870_2022_3800_MOESM7_ESM.pdf]

Figure S4

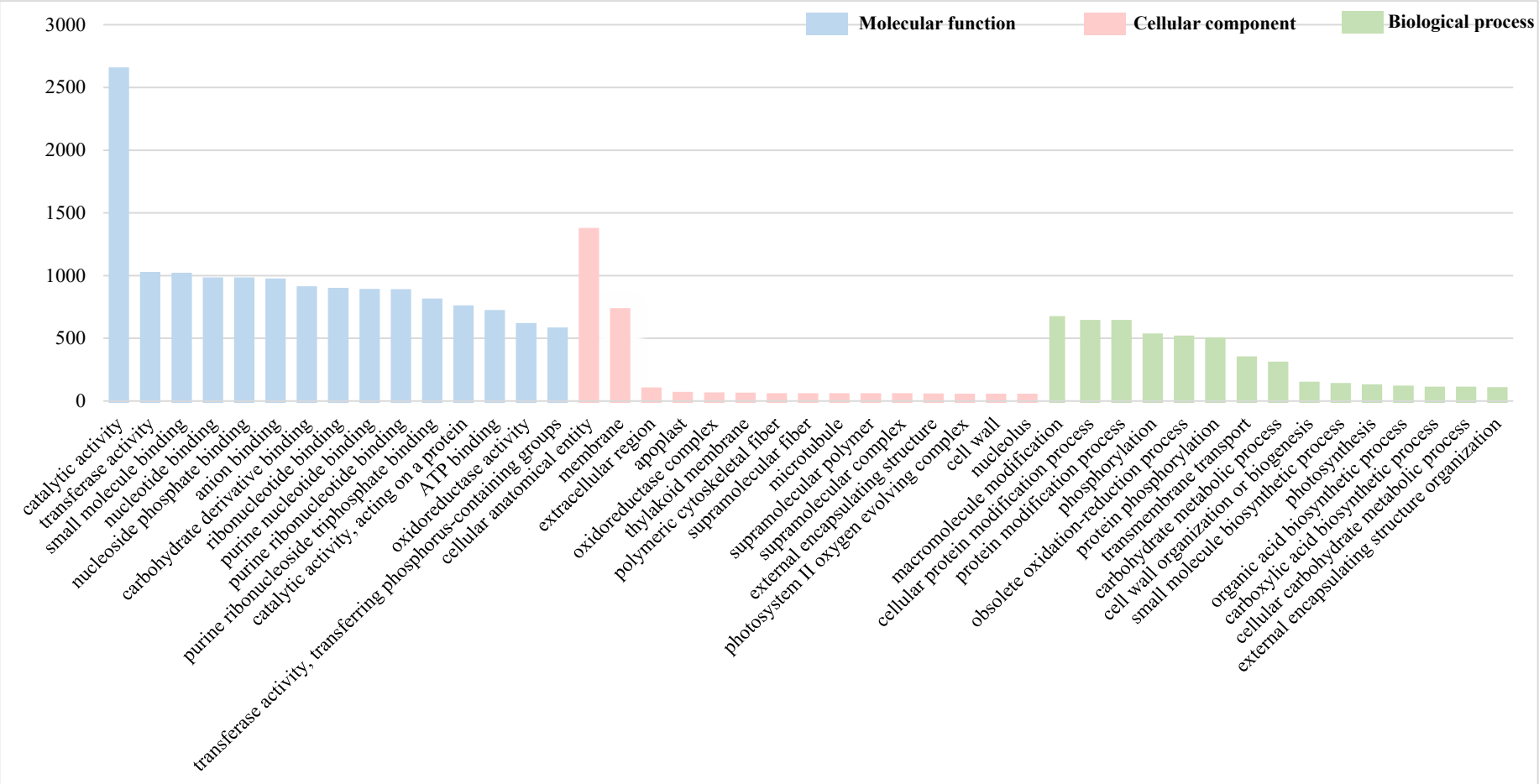

Supplement: Supplementary file 8 — Additionalfile 8: Figure S4. The enrichment results of all differentially expressed genes. [file 12870_2022_3800_MOESM8_ESM.pdf]

# Figure S5

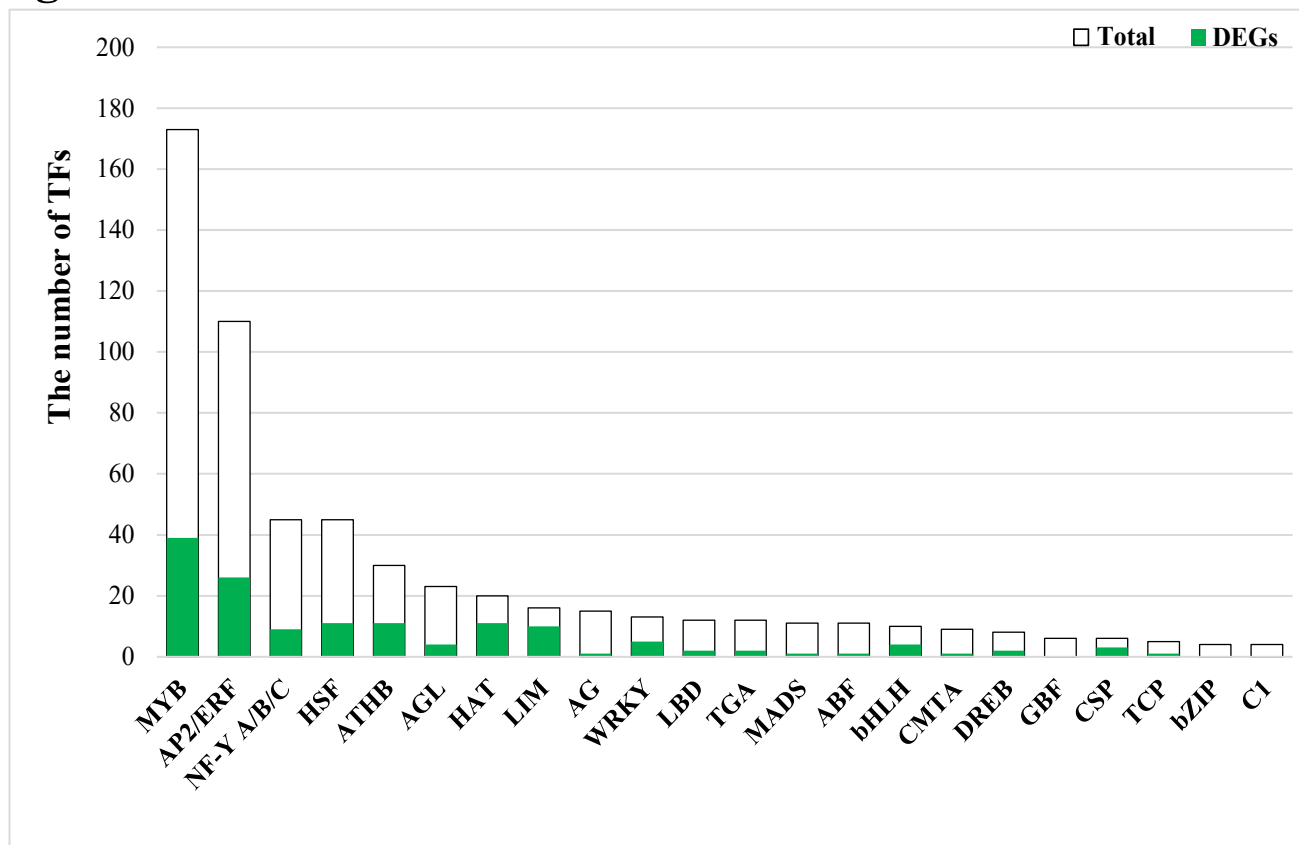

Supplement: Supplementary file 9 — Additionalfile 9: Figure S5. The identification and classification results of all TFs. [file 12870_2022_3800_MOESM9_ESM.pdf]
